# Entropic Uncertainty Relation and Information Exclusion Relation for multiple measurements in the presence of quantum memory

**DOI:** 10.1038/srep11701

**Published:** 2015-06-29

**Authors:** Jun Zhang, Yang Zhang, Chang-shui Yu

**Affiliations:** 1 School of Physics and Optoelectronic Technology, Dalian University of Technology, Dalian 116024, P. R. China

## Abstract

The Heisenberg uncertainty principle shows that no one can specify the values of the non-commuting canonically conjugated variables simultaneously. However, the uncertainty relation is usually applied to two incompatible measurements. We present tighter bounds on both entropic uncertainty relation and information exclusion relation for multiple measurements in the presence of quantum memory. As applications, three incompatible measurements on Werner state and Horodecki’s bound entangled state are investigated in details.

In quantum mechanics, there is generally an irreducible lower bound on the uncertainty in the outcomes of simultaneous measurements of noncommuting observables, i.e., the uncertainty principle which dates back to Heisenberg^1^, illustrates the the difference between classical and quantum world and forms the basis of the indeterminacy of quantum mechanics. The Heisenberg uncertainty principle originally came from a thought experiment about the measurements of the position and the momentum and later was generalized by Kennard^2^ and Robertson^3^ to arbitrary observables *X* and *Y* with a strict mathematical formulation 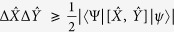 where 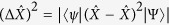 represents the variance and 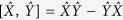 stands for the commutator. However, the standard deviation in Robertson’s relation is not always a suitable measure of uncertainty^4^,^5^. In addition, even though Robertson’s relation is good when *X* and *Y* are canonically conjugate, the right-hand side (RHS) of Robertson’s relation depends on a state 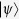, which will provide a trivial bound if 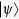 leads to the *zero* expectation value of the commutator. This kind of uncertainty relations has been studied widely in both theory^6^,^7^,^8^ and experiment^9^,^10^,^11^,^12^,^13^,^14^.

Instead of standard deviation, Deutsch^15^ quantified uncertainty in terms of Shannon entropy and derived the entropic uncertainty relation (EUR) for any pair of observables^16^. Later Maassen and Uffink^17^ improved Deutsch’s job and gave the following tighter entropic uncertainty relations:
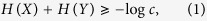
where *H*(*X*) (*H*(*Y*)) is the Shannon entropy of measurement outcomes when a measurement of observable *X* (*Y*) is performed on a state *ρ*, and 
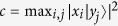
 quantifies the complementarity of the non-degenerate observables *X* and *Y* with 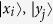 denoting their eigenvectors, respectively. It is obvious that the bound in Eq. (1) is state-independent. Hall extended the EUR given by Eq. (1) and presented an information exclusion principle which bounds accessible information about a quantum system given by an ensemble of states when two observables are performed on it^18^. The information exclusion principle for two observable *X* and *Y* and the ensemble 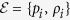 is given by
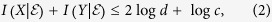
where *d* is the dimension of measurement and 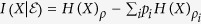 is accessible information about ensemble *ε* with *X* performed on it. Both bounds in Eqs (1,2) have been further improved to different extents^19^,^20^,^21^. The information exclusion principle and especially EUR have been studied widely^4^,^22^,^23^,^24^,^25^,^26^. It has been found that EUR has interesting applications in various quantum information processing tasks (for example^4^,^27^,^28^,^29^,^30^, and references therein). In particular, considering the direct application in quantum key distribution, Berta *et al.*^24^ generalized EUR (1) to the case in the presence of memory, that is,
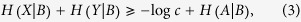
where 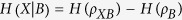 is the conditional von Neumann entropy and *H*(*ρ*) is the von Neumann entropy with *ρ*_*XB*_ denoting the state after *X* measurement on subsystem *A* of *ρ*_*AB*_ and *ρ*_*B*_ denoting the reduced state of *ρ*_*XB*_. Similarly information exclusion relation was also generalized to the case of quantum memory by replacing the classically mixing ensemble *ε* with a quantum system *B*^20^, that is,
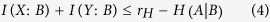
with 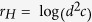. In particular, we let IER abbreviate the information exclusion relation with quantum memory implied. However, most of the relevant jobs usually consider the case of a pair of observables (measurements).

Recently, the uncertainty relations with multiple measurements have attracted increasing interests. Significant progresses have been made to seek for the uncertainty relations for more than two observables^31^,^32^, even though the uncertainty relations with two observables can automatically induce the corresponding uncertainty relations with more than two observables. In fact, among all the relevant researches, one of the most fundamental question is that the bounds are not tight enough in general or precisely speaking, are only tight for some particular states. So in this paper we would like to present the improved EUR and IER for multiple measurements in the presence of quantum memory. One will find that our bounds for EUR and IER are generally tighter than previous ones and state-independent, in particular, it can also be easily reduced to the case without quantum memory. As applications, we investigate three incompatible measurements on Werner states and Horodecki’s bound entangled states in details.

## Results

### Entropic uncertainty relation for multiple measurements in the presence of quantum memory

To begin with, let’s consider an uncertainty game between Alice and Bob similar to Ref. 32. Before the game, Alice and Bob agree on a group of measurements 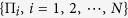 with 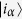 denoting *α*th eigenvector of the 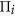. Suppose that Bob prepares a bipartite quantum state *ρ*_*AB*_ in (*d* ⊗ *d*) -dimensional Hilbert space and then sends particle *A* to Alice. Alice performs one measurement 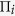 and announces her choice to Bob. Bob tries to minimize his uncertainty about Alice’s measurement outcomes.

We proceed by deriving our uncertainty relation. To do so, let’s rearrange the measurements 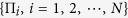 in a new order with *ε* denoting the new order. So 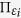 can be understood as *i*th measurement in the *ε* order. Similarly, the *α*th eigenvector of 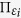 can be written as 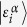. With these notations, we arrive at the following EUR for the above game in the presence of quantum memory (Proof given in Methods):
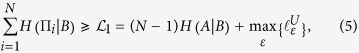
where
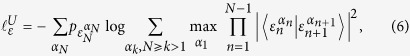
with 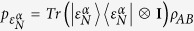. One will find that the left-hand side (LHS) of Eq. (5) quantifies the total uncertainty about the measurement outcomes, whilst the right-hand side (RHS) of Eq. (5) includes two terms. The first term 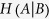 depends on the initial state and can describe the effects of entanglement on the EUR. With the entanglement of *ρ*_*AB*_ increasing, the RHS of Eq. (5) could be negative, but RHS is never negative. At this moment, Eq. (5) will reduce to a trivial form 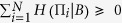. The second term 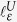 depends on the sequence of observables, the overlap of the projective measurements and the last observable’s probability distribution, it describes the measurement incompatibility.

When only two measurements 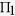 and 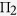 are considered, by a simple substitution, our EUR Eq. (5) becomes
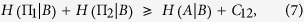
where *C*_*ij*_ = max{*C*_*ij*_,*C*_*ji*_} with 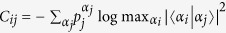. It is easy to find that this EUR is just consistent with the tight state-dependent bound of EUR improved by Coles^20^. If the state *ρ*_*AB*_ is pure, 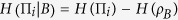 and *H*(*ρ*_*A*_) = *H*(*ρ*_*B*_)^33^. So the uncertainty relation with quantum memory for pure states *ρ*_*AB*_ can be given by
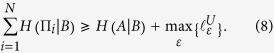


Our EUR can be easily reduced to the case without quantum memory. To do so, we substitute *ρ*_*AB*_ = *ρ* ⊗ *ρ*_*a*_ into Eq. (5), we can immediately obtain the EUR for the state *ρ* without quantum memory as
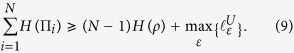


It is obvious that the probability distribution in all EUR is a function of the initial state. In order to eliminate the state-dependency, we will take maximum over *α*_*N*_ of 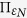, so 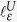 in the second term becomes
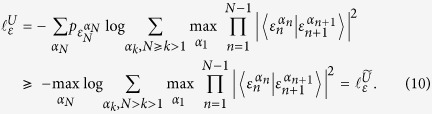


Thus, the EUR independent of state can be rewritten as
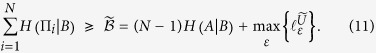


As mentioned above, the uncertainty relations for only two observables actually automatically provides an intuitive bound. Mathematically, Bob can always employ Eq. (7) (or Eq. (3)) for each possible pairs of measurements of 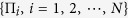, and then sum the equations in all kinds of ways and make a proper average finally, so long as he keeps 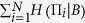 in LHS. Bob has many ways to do so and finally select the maximal one as the bound. It is formally given by
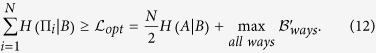
where 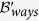 is average value of *C*_*ij*_ in Eq. (7) for all potential two-measurement combinations. For example, only one way is present for *N* = 3 and there are 7 ways for *N* = 4. Eq. (12) has consistent form with Eqs (5) and (11), which also shows the effects of entanglement between *A* and *B*. Thus we have shown two approaches to obtaining the EUR. However, one will see that neither alone can serve as a good bound in a general case. They depend the set of observables. So the tighter EUR should be summarized by collecting all the contributions (also including all the possible results that we don’t know) as
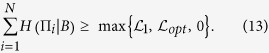


Similarly, the state-independent EUR can also be obtained easily.

### Information exclusion relation for multiple measurements in the presence of quantum memory

The IER was formulated by Hall. It looks like a transformation of the uncertainty relation based on the mutual information *I*(*A* : *B*) = *H*(*ρ*_*A*_) + *H*(*ρ*_*B*_) − *H*(*ρ*_*AB*_). Along the similar game as EUR, Alice and Bob shared a bipartite quantum system *ρ*_*AB*_. Alice performs projective measurements 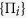 on her particle, and the particle at Bob’s hand becomes a quantum register that can record the relevant information. Thus the accessible information is bounded by the IER which is given by Eq. (4). The IER implies that the information content of quantum observables can be increased only at the expense of the information carried by complementary observable. It is just a little difference from the EUR. In particular, one notes that 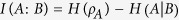. Hence we can substitute this relation into the above EURs and find the corresponding upper bounds on the mutual information, i.e., the IER. Following the completely parallel procedure as EUR, we can present our IER for multiple observables in the presence of memory as
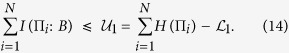


If we limit only two projective measurements 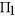 and 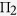, the IER will reduce to
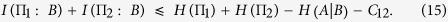


Analogous to EUR, for multiple measurements one can also select any pair of observables and use the IER given in Eq. (15). Thus one will obtain a series of equations. Keep 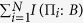 in the LHS, one will give an upper bound. Considering different combinations of the observables, one can obtain many upper bounds. We choose the minimal one as the final upper bound. Hence, such an IER can be formally given by
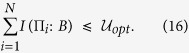


Thus the tighter bound for IER should be written as
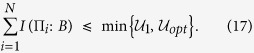


Similarly, from Eq. (16), one can obtain a state-independent upper bound denoted by 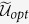. From Eq. (11), one can get the state-independent IER as
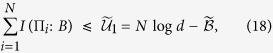
with 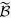 defined in Eq. (11). The IER given in Eq. (18) is obtained by taking the maximum probability 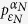. Alternatively, we can employ the concavity of the logarithm to find another bound as
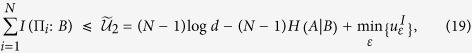
with
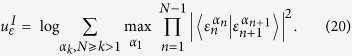


Summarizing Eq. (18) and Eq. (19) as well as 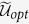, one can write the state-independent IER as
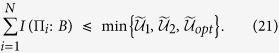


The necessary derivations of the results in Eq. (21) are given in Methods.

### Applications for three projective measurements

As applications, we first consider three two-dimensional observables measured on the Werner state which is given by^34^
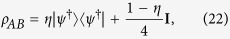
with 
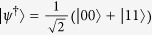
 the maximally entangled state and 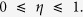 Let *X* denote an observable with the eigenvectors given by
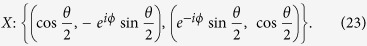


Similarly, we can define the other two observables *Y* and *Z* as follows:
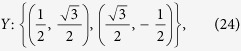

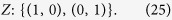


**Figure 1:**
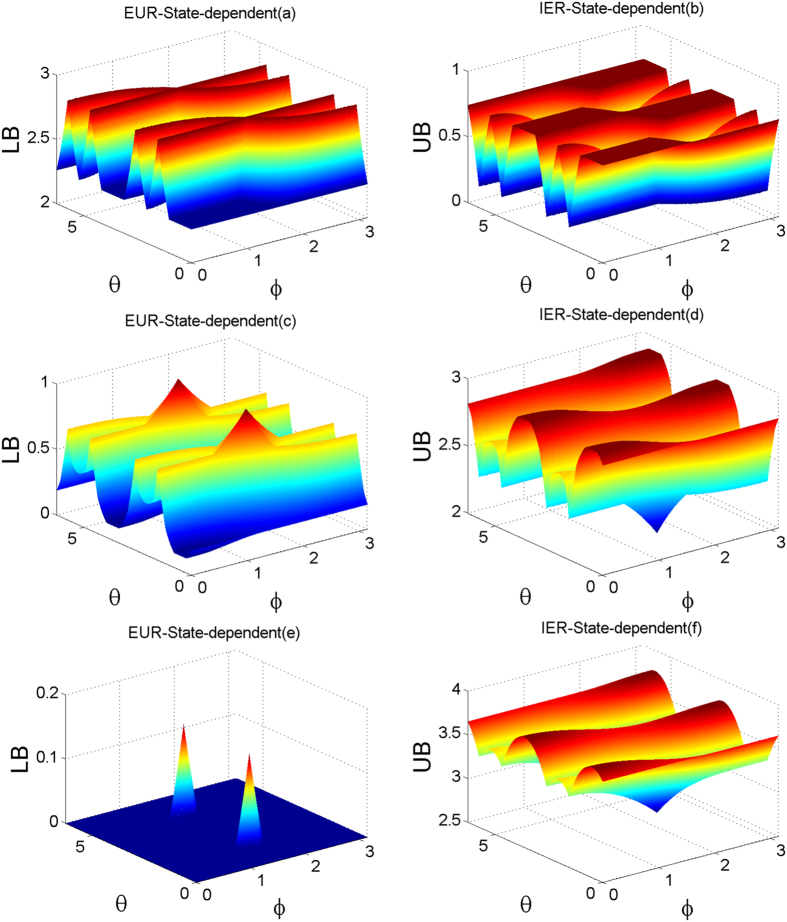
The bounds of entropic uncertainty relation and information exclusion principle for the three measurements in two-dimensional space in the presence of quantum memory vs. the azimuthal angle *φ* and the polar *θ* of the first observable. The left column (**a**,**c**,**e**) correspond to the entropic uncertainty relation and the right column (**b**,**d**,**f**) correspond to the information exclusion relation. From the top to the bottom, the purity *η* of Werner state takes 0.2, 0.8 and 0.95, respectively.

**Figure 2:**
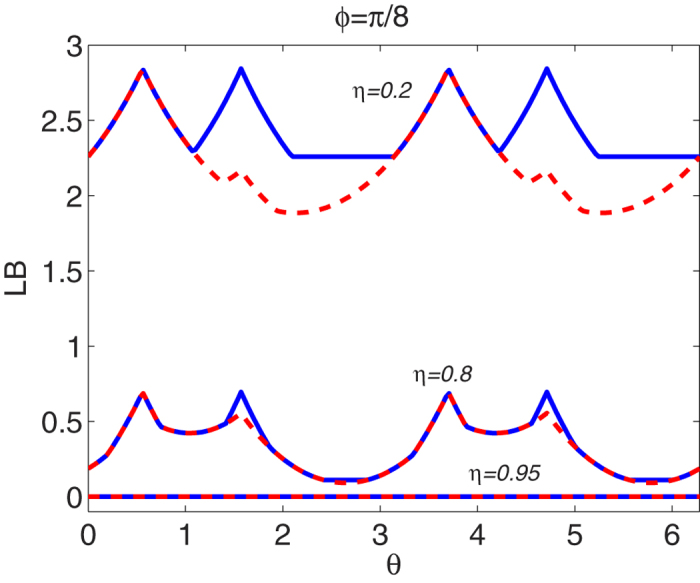
The state-dependent bounds of EUR vs. the polar *θ* when the azimuthal angle *φ* = *π*/8 of the first observable. The blue lines correspond to the state-dependent bound of entropic uncertainty relation in Eq. (13) while the red dash lines correspond to the previous one in Ref. 32. From the top to the bottom, the purity *η* of Werner state takes 0.2, 0.8 and 0.95, respectively.

As an example, we only illustrate the state-dependent EUR and IER. The bounds of EUR and IER with various purities *η* of the Werner state are plotted in Fig. 1. As we know, if the purity 0 ≤ *η* ≤ 1/3, the Werner state is separable. Figure 1(a) shows that the shape of the bounds of EUR looks like a double alphabet “X” when the Werner state includes no entanglement. However, with the purity increasing, the bounds of EUR will become small due to the generation of entanglement of the Werner state, which is given in Fig. 1(c). But the crossing point of the alpahbet “X” reduces slowly. With the purity getting much stronger, the bound of the entropic uncertainty relation is shown in Fig. 1(e) with *η* = 0.95. The crossing points of the double alphabet “X” becomes two peaks. If the purity *η* gets stronger and stronger, which means that the entanglement of the Werner state becomes much larger, the bounds of the EUR will decrease further until it goes down to 0. At that moment, the bound is trivial. The opposite behaviors can be found for the IER which are illustrated by Fig. 1(b,d,f). However, one can find that the bounds of IER is still acceptable, even though the bounds for EUR could be trivial. While in Fig. 2, we set the azimuthal angle *φ* = *π*/8 of the first observable, the blue lines correspond to the state-dependent bound of entropic uncertainty relation in Eq. (13) while the red dash lines correspond to the previous one in Ref. 32. One can find that our bound is tighter than previous one.

Next, we consider another example with three observables in three-dimensional Hilbert space. Here the measured state is the Horodecki’s bound entangled state which reads^35^.
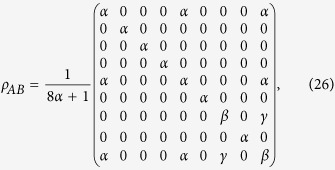
with 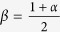 and 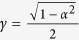. The eigenvectors for the first observable *X* is supposed to be
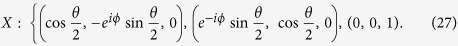


In addition, we randomly generate 3 groups of observables {*Y*, *Z*} with the eigenvectors of *Y* and *Z* given respectively by
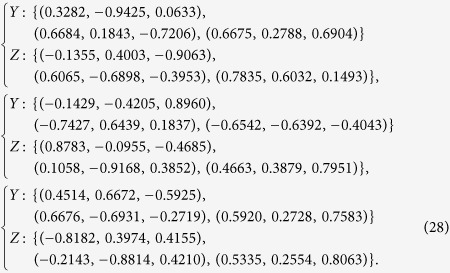


**Figure 3:**
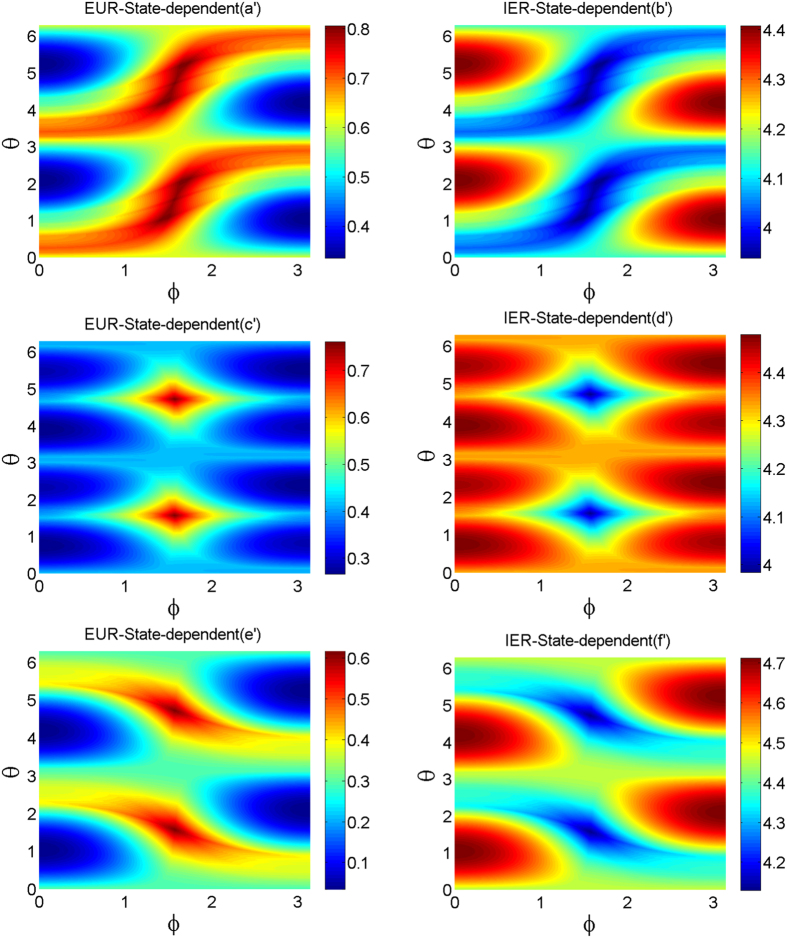
(color online) The bounds of entropic uncertainty relation and information exclusion relation for the three measurements in three-dimensional space in the presence of quantum memory vs. the azimuthal angle *φ* and the polar *θ* of the first observable. The left column (a′), (c′), (e′) correspond to the entropic uncertainty relation and the right column (b′), (d′), (f′) correspond to the information exclusion relation. In all cases, *α* = 0.6.

The bounds of EUR and IER versus *θ* and *φ* are plotted in Fig. 3. The left column in Fig. 3 corresponds to the lower bounds of EUR and the right column corresponds to the upper bounds of IER. Each row corresponds to one choice of Eq. (28). All the figures show the tightness of our bounds.

## Discussions

Uncertainty relations are the fundamental features of quantum mechanics and have wide applications in quantum information processing tasks. We have considered the EUR and IER for more than two observables in the presence of quantum memory and presented tighter bounds for them. From our results one can easily obtain the EUR in the absence of quantum memory. The nontrivial bounds of EUR and IER can be determined by the complementary of the measurements and the entanglement of the composite system. As a consequence, the nontrivial bounds shed new light on quantum uncertainty.

## Methods

Before the proof of Eq. (5), we would like first to give a lemma.

**Lemma** For a bipartite quantum system *ρ*_*AB*_ and a group of measurements 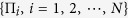 which are performed on the subsystem *A*, there will have the following relations:
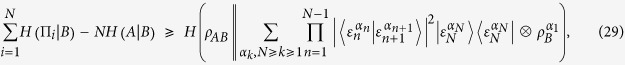
with 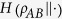 denoting the relative entropy.

Proof. First, we prove that a pair of the projective measurements 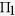 and 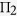 are acted on the inital quantum state, the above relation hold. That is, for *N* = 2, we have
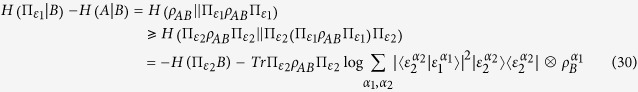

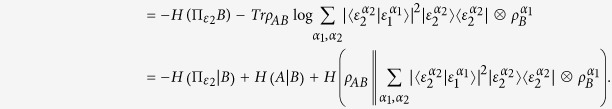


Here the inequality holds because of the adjoint concavity of relative entropy, i.e., 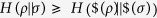 with $(·) denoting a superoperator. Thus, for a pair of measurements applied on the subsystem *A*, the following relation is satisfied:
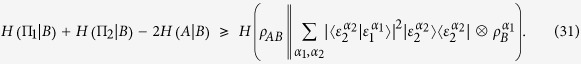


Now, let’s assume that when a set of nondegenerate measurements 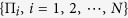 are performed on the subsystem *A*, the inequality hold for the *N* measurements. Thus, considering the set of measurements 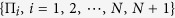, we have
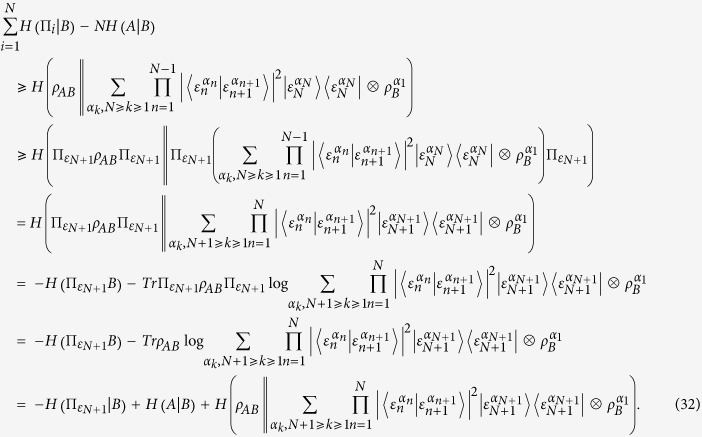


Rearrange the above inequality, we will find that
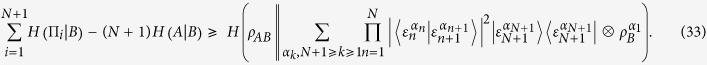


During this process, we let the first measurement 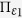 perform on the local system *A* and use 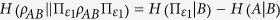. In addition, the first and the second inequalities are satisfied again due to the adjoint concavity of relative entropy. The proof of the lemma is completed.

Proof of the Eq. (5). Using the lemma, the EUR of *N* measurements can be given as follows.
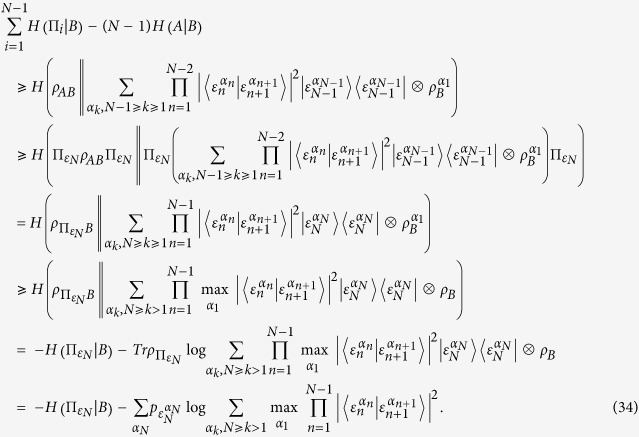


The first and the second inequality is again based on the adjoint concavity of relative entropy and the third inequality holds due to the property of the relative entropy: 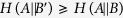, if and only if 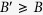. In order to find the tighter bound of the EUR, one has to find the maximum of the set 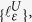 where 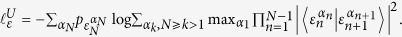 The proof is finished.

Proof of Eq. (21). From the definitions of the mutual information 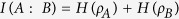 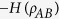
and the conditional entropy 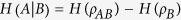, one will immediately arrive at
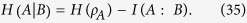


Substitute this relation into Eq. (11), we have
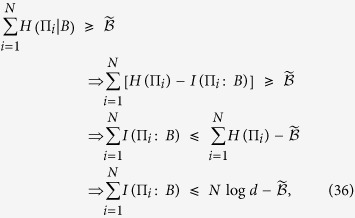
where the last inequality holds for 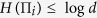.

The proof of 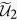. This proof can be done from Eq. (5). Substitute Eq. (35) into Eq. (5), we arrive at
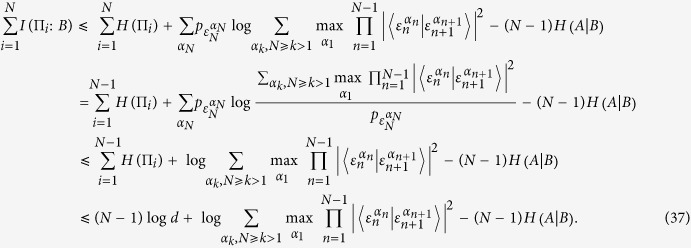


Here the second inequality is satisfied because of the concavity of the logarithm function. Similarly, in order to find the tight bound of the IER, one has to find the minimum of the set 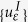 with 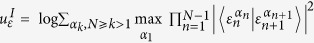.

## Additional Information

**How to cite this article**: Zhang, J. *et al.* Entropic Uncertainty Relation and Information Exclusion Relation for multiple measurements in the presence of quantum memory. *Sci. Rep.* **5**, 11701; doi: 10.1038/srep11701 (2015).
